# Primary Malignant Melanoma of the Cervix: An Integrated Analysis of Case Reports and Series

**DOI:** 10.3389/fonc.2022.913964

**Published:** 2022-06-22

**Authors:** Aiping Min, Aizhen Fu, Meiyuan Huang, Hongjing Wang, Huan Chen

**Affiliations:** ^1^ Department of Obstetrics 1, Zhuzhou Central Hospital, Zhuzhou, China; ^2^ Department of Obstetrics and Gynecology, People’s Hospital of Leshan, Leshan, China; ^3^ Department of Obstetrics and Gynecology, Affiliated Hospital of Guangdong Medical University, Zhanjiang, China; ^4^ Department of Pathology, Zhuzhou Central Hospital, Zhuzhou, China; ^5^ West China Second University Hospital, Sichuan University, Chengdu, China

**Keywords:** primary malignant melanoma, cervix, integrated analysis, case report, case series

## Abstract

Melanoma, also known as malignant melanoma, is a type of malignant tumour that originates from melanocytes in the basal layer of the epidermis. Primary malignant melanomas of the female genital tract are rare. Similarly, primary malignant melanoma of cervix, which originates from cervical melanocytes, is an extremely rare disease and the second most common type of female melanoma in women aged between 15 to 44 years worldwide. To date, primary malignant melanoma of the cervix is characterized by poor patient prognosis and little consensus exists regarding the best treatment therapy. The situation is worsened by lack of clinical studies with large samples. Notably, surgery remains the preferred treatment option for patients with primary malignant melanomas of the cervix. Current treatments are based on Federation International of Gynecology and Obstetrics(2018) staging with reference to National Comprehensive Cancer Network guidelines. This study is in order to find a more suitable treatment modality for primary malignant melanoma of cervix. Therefore, we first conducted an integrated analysis of case reports and series to assess the impact of various factors on the prognosis of such patients. In summary, this is the first pooled analysis including 149 cases of primary cervical melanoma. We found that patients who underwent radical hysterectomy-based surgery, those with non-metastatic lymph nodes and those who underwent lymphadenectomy had significantly higher survival rates. In patients who had RH-based surgery, survival rates at the 24m time point of those who did not add other treatments was higher than those who did, but for those who had total hysterectomy-based surgery, the addition of other treatments to prolong median survival may be considered. In the overall analysis, age and lymphadenectomy were associated with increased and reduced risk of death in these patients, respectively. Although there is no statistical difference, stage III&IV, TAH, lymphatic metastases increase the risk of death; whereas radical hysterectomy was associated with reduced risk of death. In the subgroup analysis, for patients who have undergone radical hysterectomy-based surgery, lymphadenectomy reduces the risk of death, while lymphatic metastases and complementary other treatments increase the risk of death. For patients who have undergone total hysterectomy-based surgery, complementary treatment reduces the risk of death. In conclusion, *via* summarizing previous reports, the recommended treatment procedure for PMMC are radical hysterectomy and lymphadenectomy. The addition of other treatment options for patients who undergoing RH-based surgery need further study.

## Introduction

Melanoma, also known as malignant melanoma (MM), is a type of malignant tumour that originates from melanocytes in the basal layer of the epidermis (1). It is considered one of the most aggressive cancer diseases due to its high malignancy and treatment resistance (2). Notably, MM patients have a survival period of less than 5 years (3), although they have very good prognosis if the disease is detected in its early stages (4). Globally, disease accounts for approximately 0.03% of all newly diagnosed cancers (5). Previous studies have shown that the high mortality rate is due to the aggressive metastatic potential of melanoma cells (6). Previous estimates have shown that approximately 132,000 and 48,000 new malignant melanoma cases and deaths, respectively, occur each year (7). In fact, MM is ranked the fifth most common cancer in the world, and its incidence is on the rise (8). Primary MMs of the female genital tract are rare, accounting for only 3-7% of all mucosal melanomas (9). On the other hand, primary malignant melanoma of the cervix (PMMC) is an extremely rare disease that originates from cervical melanocytes (10). It is the second most common type of female melanoma in women aged between 15 to 44 years worldwide (11).

Melanoma is a highly malignant tumor with a poor prognosis (12), while that of PMMC worse (13). To date, little consensus has been reached regarding the best treatment therapy for PMMC. Lack of clinical studies involving large sample sizes has also contributed to scarcity of optimal treatment options. Nevertheless, surgery remains the preferred treatment option (13, 14). The current treatments are based on FIGO staging with reference to NCCN guidelines (15). This study is in order to find a more suitable treatment modality for PMMC. Therefore, we first conducted an integrated analysis of case reports and series to assess the impact of various factors on the prognosis of such patients.

## Methods

### Data Sources and Search Strategy

In this section, we describe a comprehensive analysis based on published case report data. Briefly, we searched three public databases, namely PubMed, EMBASE and the Cochrane Library, for case reports published from their inception until 31st January 2022. The search strategy involved the following terms: (((Cervixes) OR (Uterine Cervix)) OR (Cervix, Uterine)) OR (Cervix)) AND (Primary malignant melanoma). In addition, we performed a secondary search of the reference lists across relevant articles to identify additional eligible reports. Two independent reviewers (Aiping Min and Aizhen Fu) conducted the literature search, study selection and data extraction. Any discrepancy between them was resolved through consensus and arbitration by a third author (Meiyuan Huang).

### Study Selection Criteria

All studies describing primary malignant melanoma of the cervix, with clinical information on the patients regardless of sample size, were included in this study. Conversely, articles that met the following criteria were excluded from the study: Primary melanoma of other genital tract origin, genital tract melanoma of undeterminable origin, reviews, books, as well as irrelevant. In cases where studies had overlapping data, we selected the study with the larger sample size.

### Data Extraction

The following data were recorded: year of publication, patient’s age at diagnosis, patient’s symptoms, FIGO stage, whether surgery was performed, mode of surgery, presence or absence of lymphatic metastases, use of other treatment modalities and overall survival (OS) in months. If the patient’s clinical information was mentioned in another article, and the corresponding full text was not available, we marked the survival status with UNCERTAIN.

### Outcomes and Statistical Analysis

Outcomes assessed were age and OS. The definition for survival time was based on data from the individual participants reported. Missing and unidentifiable data were specified as NA, thus were not included in the statistical analysis. Categorical data were presented as frequencies and percentages. On the other hand, continuous data that conformed to normal distribution were presented as means and standard deviations (SD), while non-normally distributed data were presented as medians (range). In addition, Kaplan-Meier survival curves were generated to estimate OS of patients, and differences across subgroups compared using the Log-rank test. Statistical analysis was performed using the SURVIVAL package implemented in R language (16). A multivariate analysis of these predictors of survival for overall and subgroups was conducted using a Cox proportional risk model. Data followed by P<0.05 were considered statistically significant.

## Results

### Search Results and Eligible Studies

**Figure 1:**
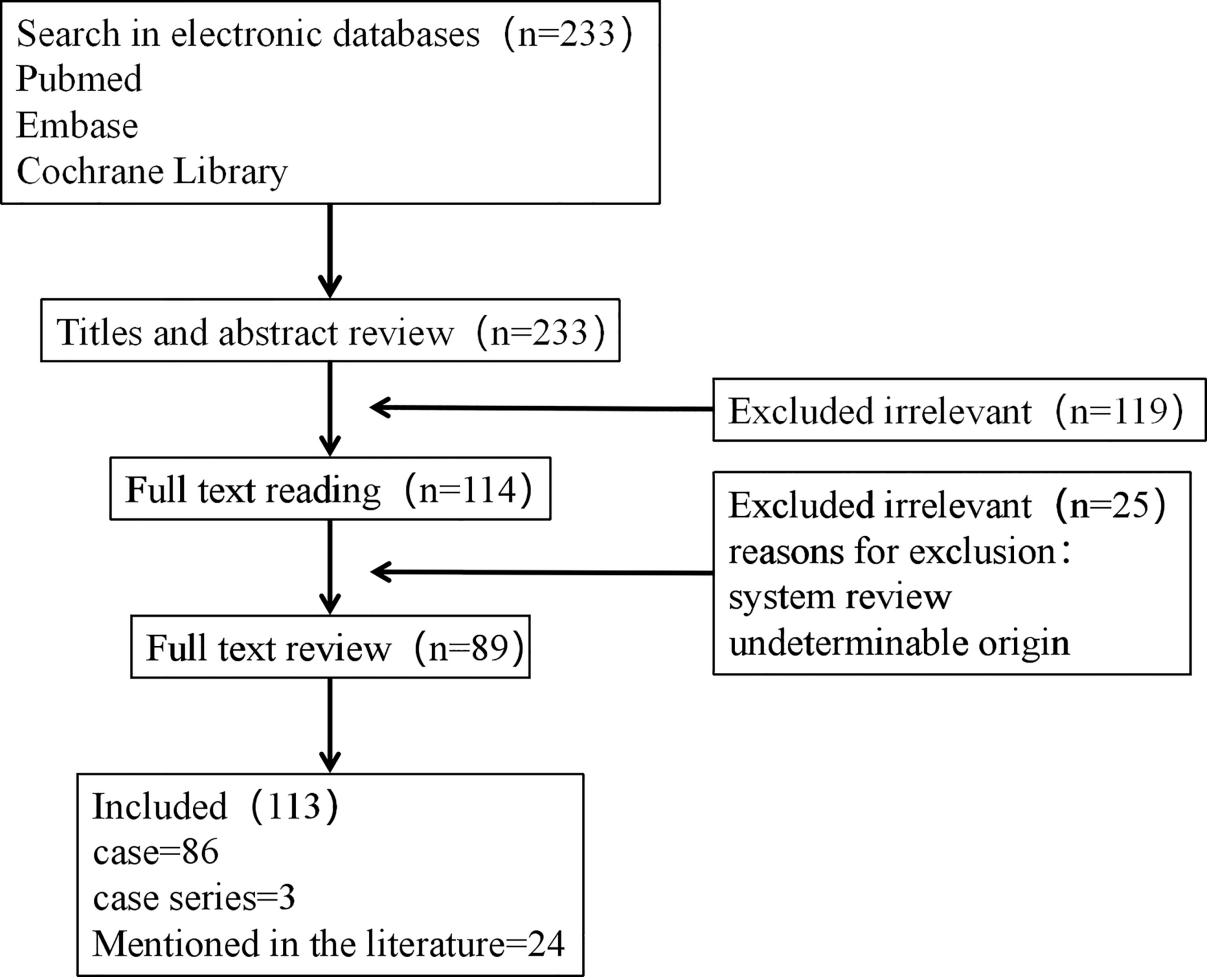
Flowchart of the study.

The aforementioned search strategy resulted in a total of 233 articles across the screened databases. Finally, 113 articles, containing 149 cases, were found to be eligible and therefore included in the analysis. A summary of the main features of the included studies is outlined in **Table 1**, whereas the procedure for literature search and selection is shown in **Figure 1**.

**Table 1:** Main features of the included studies..

Authors	Age	Symptom	Figo	Surgery	Lymph	Treatment	Survival(m)	Status
1889 (17)	40	NA	NA	yes not defined	NA	NA	NA	NA
1923 (18)	NA	VB	IIB	NA	NA	no	0.1	uncertain
1944 (19)	64	VB, A pain	IB	TAH + PV	NA	no	156	uncertain
1950 (20)	62	VB	IB	TAH	NA	R	1.2	uncertain
1954 (21)	NA	NA	NA	NA	NA	NA	NA	NA
1954 (22)	NA	VB and VD	IIIB	TAH + LND	NA	R	21	uncertain
1959 (23)	68	Post coital VB and VD	IB	LE	NA	R	23	uncertain
1959 (24)	49	Post coital VB and VD	IIIA	No	NA	R	11	uncertain
1961 (25)	64	UK	IB	TAH + BSO	NA	no	UK	NA
1961 (26)	46	Asymptomatic	IIB	No	yes	R	6	1
1966 (27)	50	VB and VD	IB	TAH + LND	NA	R	21	uncertain
1967 (28)	72	VB and VD	IB	No	NA	R	UK	NA
1967 (29)	57	VB	IIA	TAH + BSO	NA	R	12	uncertain
1970 (30)	69	Dyspareunia	IB	LE	NA	R	62	uncertain
1970 (31)	72	VD	IIIA	LE + VULV	NA	R	24	uncertain
1971 (32)	39	Asymptomatic	IIA	TAH + BSO + PLND + PV + VULV	no	C+R	168	1
1976 (33)	70	VB and VD	IIB	TAH + BSO	NA	C+R	10	1
1979 (34)	44	VB	IB	TAH + BSO	NA	I	12	0
1979 (35)	62	VB	IB	TAH + BSO	NA	C+R	19	uncertain
1979 (36)	48	UK	NA	TAH + BSO + LND	NA	C	14	uncertain
1980 (37)	26	VB and VD	IIB	Total pelvic exenteration	no	no	11	0
1981 (38)	65	VB	IIB	RH+PLND	yes	C	10	1
1981 (39)	45	VB	IIA	RH+PLND+PV	yes	no	UK	NA
1981 (40)	52	VB	IB	TAH+BSO+PV	NA	no	18	1
1986 (41)	74	VB	IIA	RH+BSO+PLND+PV	no	no	UK	NA
1987 (42)	46	VB and VD	IV	LE	NA	R	5	uncertain
1988 (43)	47	VB	IIIA	No	NA	C+R	UK	NA
1988 (44)	58	VB	IIIB	No	NA	R	9	1
1989 (45)	52	VB	IB	TAH+PLND+PV	no	R	25	1
1989 (46)	20	VD	IV	LE	yes	C	5	1
1990 (47)	64	VD	IB	RH+BSO+PLND	no	R	48	0
1990 (48)	71	UK	IB	TAH+VULV+LND	no	no	UK	NA
1990 (49)	35/58	VB/VB	IB/IIA	RH+BSO+PLND	no/no	no	17//5	0//0
1991 (50)	30	VB and A pain	IB	RH+BSO+PLND+PV	yes	R	34	0
1991 (51)	62/60/37	VB, VB, VB	III/III/IB	no/RH/RH	NA	C/C+R/no	14//12//10	1//1//0
1992 (52)	83	VB	IIIB	No	NA	R	15	1
1992 (53)	72	VB	IV	TAH+BSO	NA	C+R	12	0
1992 (54)	70	VB	IIIA	TAH+LND+VAG	NA	no	UK	NA
1993 (55)	NA	Asymptomatic	IA	RH + BSO + LND + PV	NA	R	65	uncertain
1994 (56)	70	VB	IB	RH	NA	no	18	uncertain
1995 (57)	78	VB	IB	TAH+BSO+LND	NA	no	27	uncertain
1996 (58)	72	VB	IB	LE	NA	R	UK	NA
1997 (59)	19	NA	NA	RH+BSO+PLND+PV	NA	C	UK	NA
1997 (60)	57	VB	IIA	TAH+BSO+PLND	no	C	18	1
1998 (61)	65	VB, A pain	IIIB	No	NA	R	6	1
1998 (62)	NA	VB	IIB	TAH+BSO+LND+PV	NA	no	48	uncertain
1998 (63)	51	VB	NA	TAH+BSO	NA	C+I	13	1
1998 (64)	70	VB	IIA	RH+BSO+PLND+PV	NA	R	29	0
1999 (65)	NA	VB VD Hematuria	IIB	NA	NA	R	9	uncertain
1999 (66)	76	VB	IB	RH+BSO+PLND	no	no	30	0
1999 (67)	71	VB	IVB	RH+BSO+PLND	no	C	2.75	1
1999 (68)	70	VB	IIA	RH+BSO+PLND+PV	yes	R	29	1
1999 (69)	63	VB	IB	TAH+BSO	NA	C	10	1
2000 (70)	73	VB VD	IIB	No	NA	R	8	1
2001 (71)	33	VD, A pain	IIB	RH+BSO+PLND	yes	no	6	0
2001 (72)	31	VB	IIA	TAH+BSO+LND	no	no	10	1
2001 (73)	50	VB	IB	RH+PLND	no	R	24	0
2002 (74)	70	VB, A pain	IB	RH+PLND	NA	no	6	1
2003 (75)	26//70	VB, and asymptomatic	IIIA/IB	TAH+salpingorectomy/residual cervix+PV+PLND	NA/no	I/no	11//5	0//0
2003 (76)	67	VB	IIA	TAH+BSO+PLND+PV	no	no	12	1
2003 (77)	70	VB	NA	LE	NA	C	4	1
2004 (78)	NA	VD	NA	NA	NA	NA	NA	NA
2005 (79)	39	VB, A pain	IIIB	RH+BSO+PLND+PV	yes	C+R	6	1
2005 (80)	82	NA	NA	NA	yes	R	NA	NA
2005 (81)	45*	Post coital VB and VD	NA	RH+PLND/RH+PLND/RH+PLND/RH+PLND	NA	C+R/C/no/C+I	42//42//6//84	0//1//1//0
2005 (82)	54	VB	IIA	RH	NA	C+R	8	1
2006 (83)	38	VB	IB1	RH+BSO+PLND	no	R	24	0
2007 (84)	63	Asymptomatic	NA	Residual cervix+PLND	no	no	12	0
2008 (85)	43	VB and VD	IIB	TAH+BSO+PLND + VAG	no	C	65	1
2009 (86)	40	VD	IIA	RH+PLND	yes	C+R	18	0
2009 (87)	61	VB	IB	RH+BSO+PLND	no	no	10	0
2009 (88)	58	VB	IIB	RH+PLND	NA	C	NA	NA
2009 (89)	61	vaginal spotting	IB1	RH+BSO+PLND	no	no	120	0
2009 (90)	67	VB	IIA	TAH+BSO+PLND+PV	no	C+I	6	0
2009 (91)	40	NA	NA	TAH	NA	NA	NA	NA
	61	NA	NA	TAH	NA	NA	NA	NA
2010 (92)	65	VB	IB1	no	NA	NA	NA	NA
2010 (93)	72	NA	NA	yes not defined	NA	R	NA	NA
	50	Contact VB	NA	yes not defined	NA	R	NA	NA
2010 (94)	34	VB	IV	total pelvic exenteration	yes	R	96	0
2011 (95)	75	VB	IB1	RH+BSO+PLND	NA	C+R	5	1
2011 (96)	67	VB	IB1	RH+BSO+PLND+PV	no	no	NA	NA
2012 (97)	76	VB	NA	TAH+BSO+PLND	yes	NA	NA	NA
2012 (98)	66	VB	IB1	RH+PLND+TV	yes	R	NA	NA
2013 (99)	63	AVD and occasional VB	IIA1	RH+BSO+PLND+PV	no	no	40	0
2013 (100)	35	AVD and irregular VB	IIA	TAH+PLND	yes	no	6	1
2014 (101)	35	VB and abdominal pain	IB1	NA	NA	NA	NA	NA
2014 (13)	46	VB	IB2	RH+BSO+PLND	yes	C+R	24	0
2014 (102)	65	VB	IB1	RH+BSO+PLND	no	C	30	0
2014 (103)	49	VB	IIA2	TAH+BSO+PLND	no	NA	NA	NA
2014 (104)	42	VB	IIIB	no	yes	R	5	1
2014 (105)	51	VB	IIB	TAH+BSO+PLND	no	C	10	0
2015 (106)	43	VB	IB1	RH+PLND	no	R+I	20	0
2015 (107)	43	VB	IB1	RH+BSO+PLND	NA	NA	NA	NA
2015 (108)	73	VB	IB2	RH+BSO+PLND	no	C+I	7	1
2016 (109)	61	VB	IB1	RH+BSO+PLND	no	NA	16	1
2016 (110)	70	left thigh pain	IV	no	yes	argon laser	2	1
2016 (111)	68	VB	IB2	RH+BSO+PLND	no	no	60	0
2017 (112)	47	AVD	IB2	RH+PLND	yes	C+R	NA	NA
2017 (113)	64	VB and dyspareunia	IIB	RH+PLND	no	R	21	0
2017 (114)	66	VB	IIIA	RH+BSO+PLND+TV	no	C+PD	13	1
2017 (115)	61	VB	IIB	RH+PLND	NA	I	193	0
	74	VB	IB2	RH+PLND	NA	C	33	1
	56	VB	IIIB	No	NA	C+R	5	1
	74	VB	IIA1	TAH+PLND	NA	C+I	28	1
	77	VB	IB1	TAH	NA	no	25	1
	45	VB	IB1	RH+PLND	NA	no	87	0
	50	VB	IB2	TAH+PLND	NA	C	16	1
	58	VB	IB1	RH+PLND	NA	C	35	0
	57	VB	IIA1	No	NA	C	4	1
	42	VB	IIB	TAH	NA	R	9	1
	63	VB	IIIB	No	NA	C+R	10	1
	54	VB	IIB	RH+PLND	NA	C+R	33	1
	78	VB	IIIB	No	NA	C+R	12	1
	68	VB	IIA1	RH+PLND	NA	no	20	1
2018 (116)	56	VB	IB1	RH+BSO	no	C	36.5	1
	62	VB	IIB	RH+BSO	yes	C+R+I	13.7	1
	38	VB	IVB	No	yes	no	UK	NA
	62	VB	IB1	RH+BSO	NA	no	70	0
	53	VB	IB1	RH+BSO	NA	Argon heliumknife +Sunitinib	51.5	1
	57	VD	IIB	No	NA	C+I	6	1
	80	VB	IIIB	No	NA	no	3	1
	54	VB	IB1	RH+BSO	NA	no	UK	NA
	50	VB	IIA2	TAH	Yes	no	UK	NA
	58	VB	IIA	TAH	NA	R	20	1
	45	VB	IB1	RH+BSO+PLND	Yes	C	3	1
	55	VB	IIB	RH+BSO	NA	C+R	5	1
	60	VB	IB1	RH+BSO	No	no	5	1
	69	Urinary incontinence	IB1	RH+vulva+local urethal resection	NA	C+I	16	1
2018 (117)	47	Contact bleeding	NA	RH	NA	no	NA	NA
2018 (118)	40	VD	IIA	RH+BSO+PLND+PV	yes	PD	8	1
2018 (119)	42	VD	NA	TAH+BSO	NA	C	18	0
2019 (120)	54	Abnormal cervical cytology	IB1	RH+BSO+PLND	no	PD	50	0
2019 (121)	55/81	VB	IIA/NA	RH+BSO+PLND/no	NA/NA	C/no	67//21	0//0
2020 (122)	34	annual examination	IB1	RH+BSO+PLND	no	PD	13	1
2020 (123)	25	acute pain in the lower abdomen	IVA	RH+PLND	yes	oncolytic virus Rigvir^®^	67	0
2020 (124)	68	VB	NA	NA	NA	NA	NA	NA
2020 (125)	28	NA	IIB	RH+PLND	NA	NA	NA	NA
2021 (126)	74	VB	IB1	RH+BSO	no	PD	6	1
2021 (127)	68	VB	II	TAH+BSO+PLND	no	no	3	1
2022 (128)	73	VB	IIA1	RH+BSO+PLND	Yes	R+PD	7	1

VB, vaginal bleeding; VD, vaginal discharge; RH, radical hysterectomy; TAH, total hysterectomy; BSO, bilateral salpingo-oopherectomy; LND, lymph node dissection; PLND, pelvic lymph node dissection; PV, partial vaginal excision; LE, Local excision; C, chemotherapy; R, radiotherapy; I, immunotherapy(interferon-γ or interleukin-2); PD, PD-1/PD-L1; Others: oncolytic virus Rigvir^®^,argon laser,Argon heliumknife+Sunitinib.

NA, Not Available

### Patients’ Clinicopathological Characteristics

A total of 149 patients with PMMC were recruited in this study. Seven cases with missing data were excluded. The remaining cases had a median age of 58 years at diagnosis. Among the 149 patients, 126 (126/149) were suffered from vaginal bleeding or vaginal discharge, 2 due to contact bleeding, 7 asymptomatic, one each for acute pain in the lower abdomen, dyspareunia, left thigh pain, urinary incontinence, and 11 because of unknown reasons. FIGO staging guidelines stratified 56 patients into stage I, 45 into stage II, 18 into stage III, 8 in stage IV and 22 for whom staging information was not available. Notably, 7 patients had no surgical information, while 20 had no surgery and 7(4.6%) only had local excision. On the other hand, 77.1% (115/149) of patients were treated with surgery, 46.9%(70/149) patients had surgery based on radical hysterectomy (RH), 2 of whom had total pelvic exenteration, 28.2%(42/149) patients had surgery based on total hysterectomy (TAH), while 3 had surgery but not defined at all. In addition, 25 and 38 exhibited presence and absence of lymph node metastases, respectively, while corresponding information was not available in the remaining patients. For the 25 patients with lymph node metastases, lymphadenectomy was performed in 17 cases. Furthermore, 79 and 60 patients underwent and did not undergo lymphatic resection, respectively, with the rest of the cases lacking corresponding information. Finally, 68 patients received whereas 37 did not receive treatment other than surgery, which included radiotherapy, chemotherapy, immunotherapy(interferon-γ or interleukin-2), PD/PD-1 inhibitors, oncolytic virus Rigvir^®^, argon laser, Argon heliumknife. Among the 68 patients, 18 patients received chemotherapy, 6 patients received chemotherapy+immunotherapy, 1 patients received chemotherapy+PD, 14 patients received chemotherapy+radiotherapy,1 patients received chemotherapy+radiotherapy+immunotherapy,3 patients received immunotherapy, 2 patients received others(oncolytic virus Rigvir^®^, argon laser, Argon heliumknife) treatment,4 patients received PD, 17 patients received radiotherapy, 1 patients received radiotherapy+immunotherapy, 1 patients received radiotherapy+PD.

### Patient Prognosis and Survival Rates

**Figure 2:**
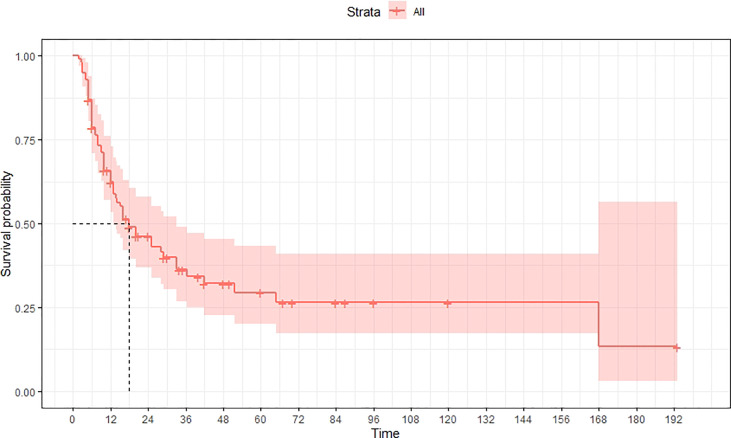
Kaplan–Meier survival curves for OS of patients. The median OS was 18 months.

**Figure 3:**
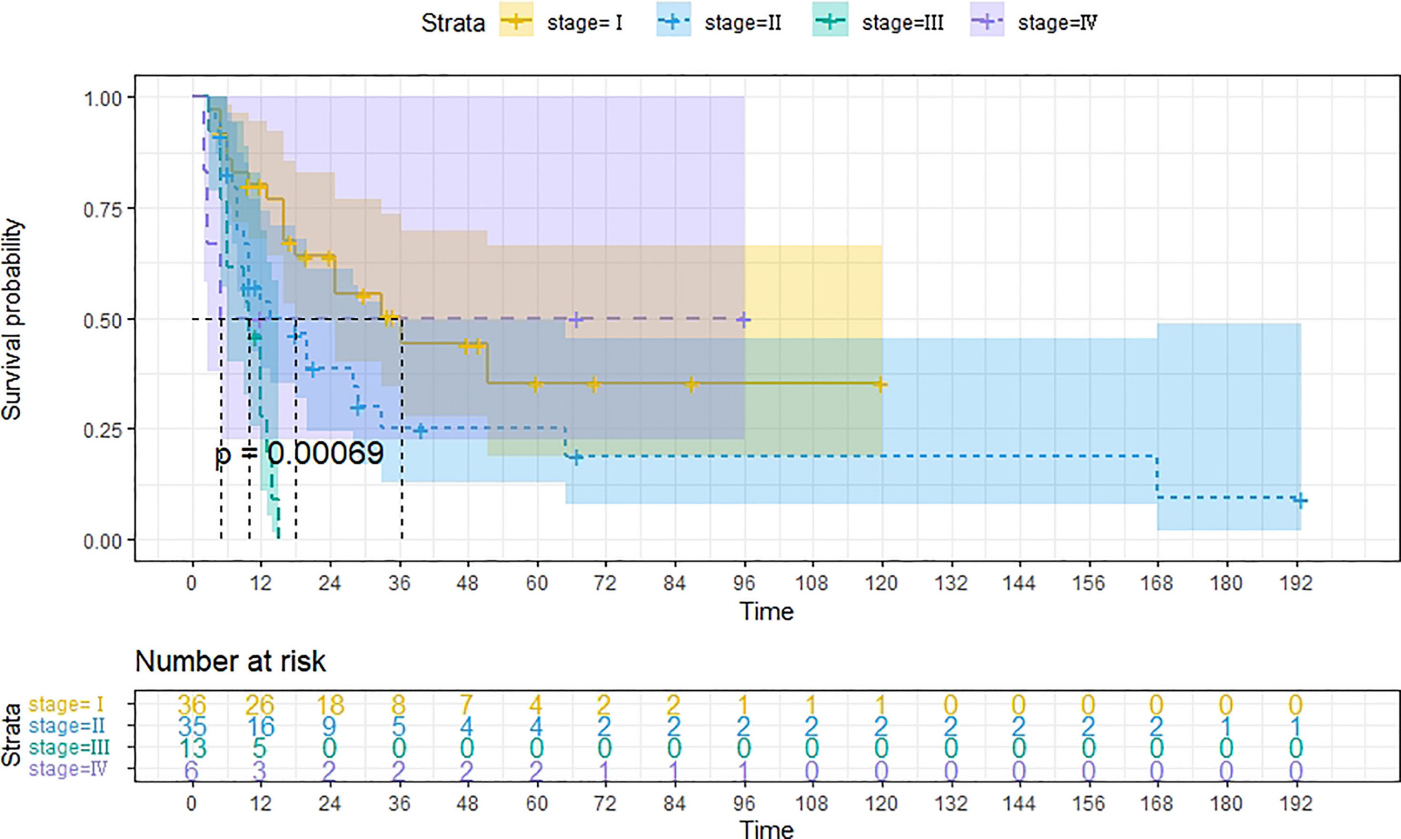
Kaplan-Meier survival curves for OS in patients of different FIGO stage. The median OS for FIGO I, II, III, IV are 36m, 18m, 10m, 5m respectively.

**Figure 4:**
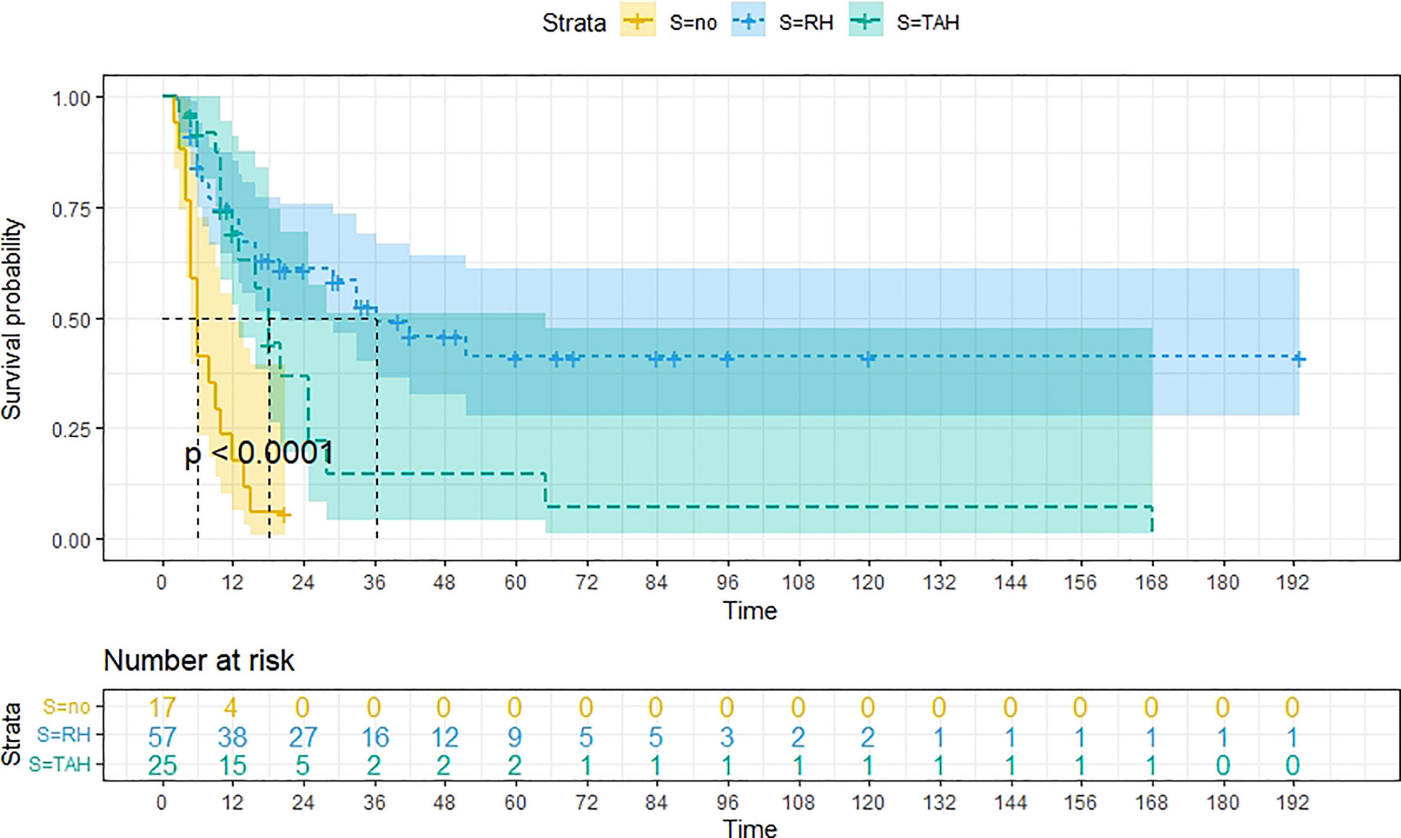
Kaplan-Meier survival curves for OS in patients of no surgery, TAH-based surgery, RH-based surgery. The median OS for them are 6m, 18m, 36m, respectively.

**Figure 5:**
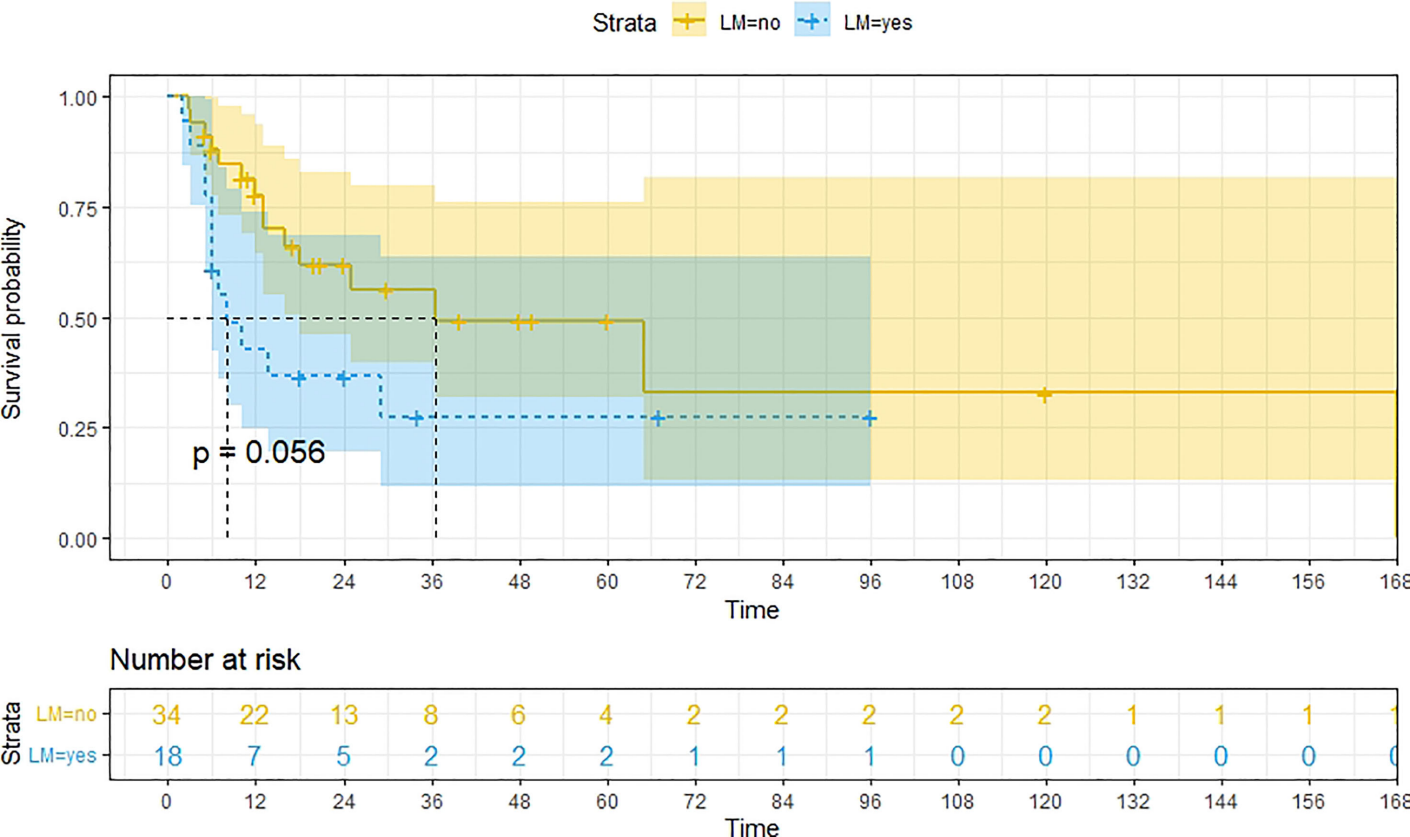
Kaplan-Meier survival curves for OS in patients of no lymphatic metastases and Lymphatic metastases. The median OS for them are 37m, 8m respectively.

**Figure 6:**
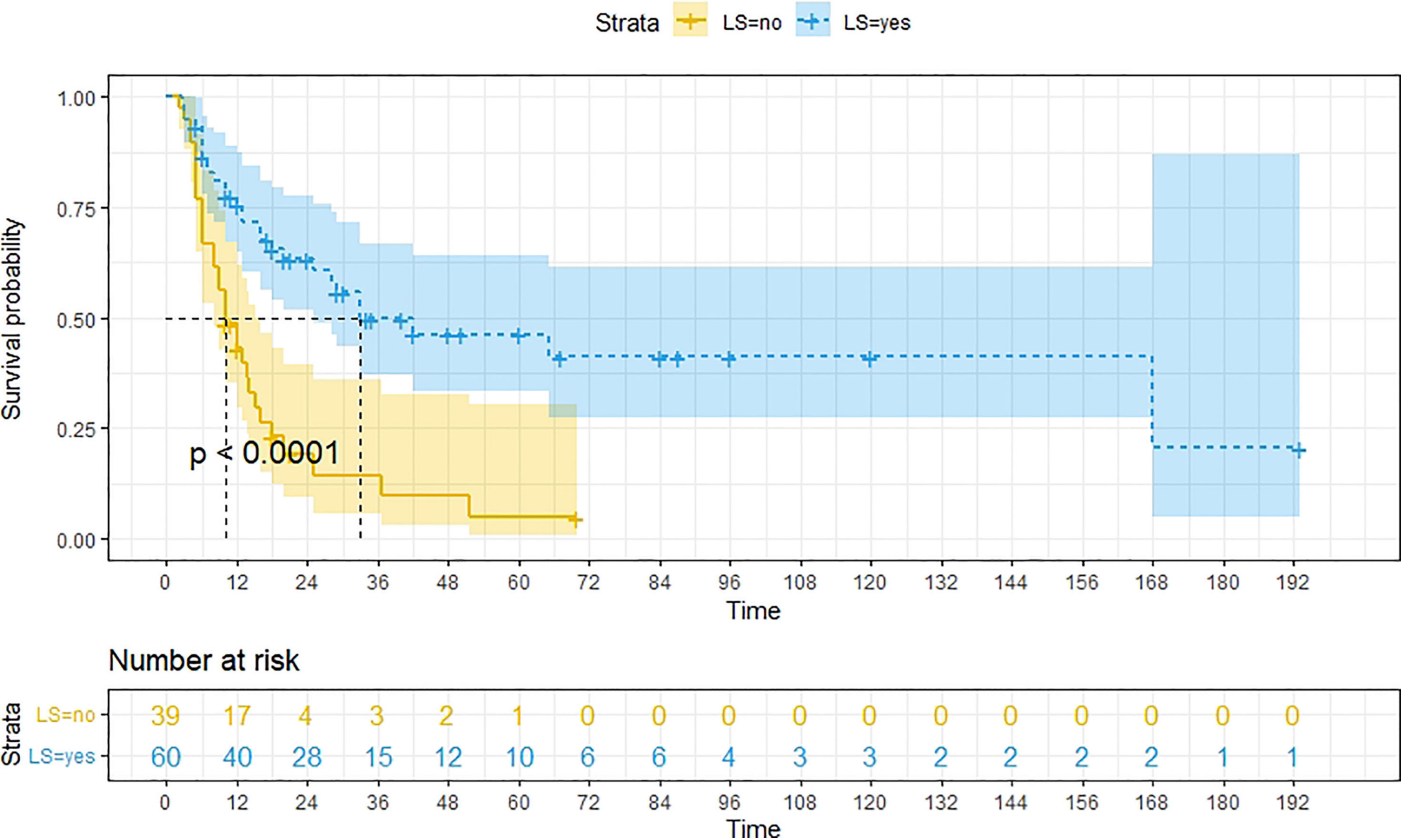
Kaplan-Meier survival curves for OS in patients of no Lymphadenectomy and Lymphadenectomy. The median OS for them are10m, 33m respectively.

**Figure 7:**
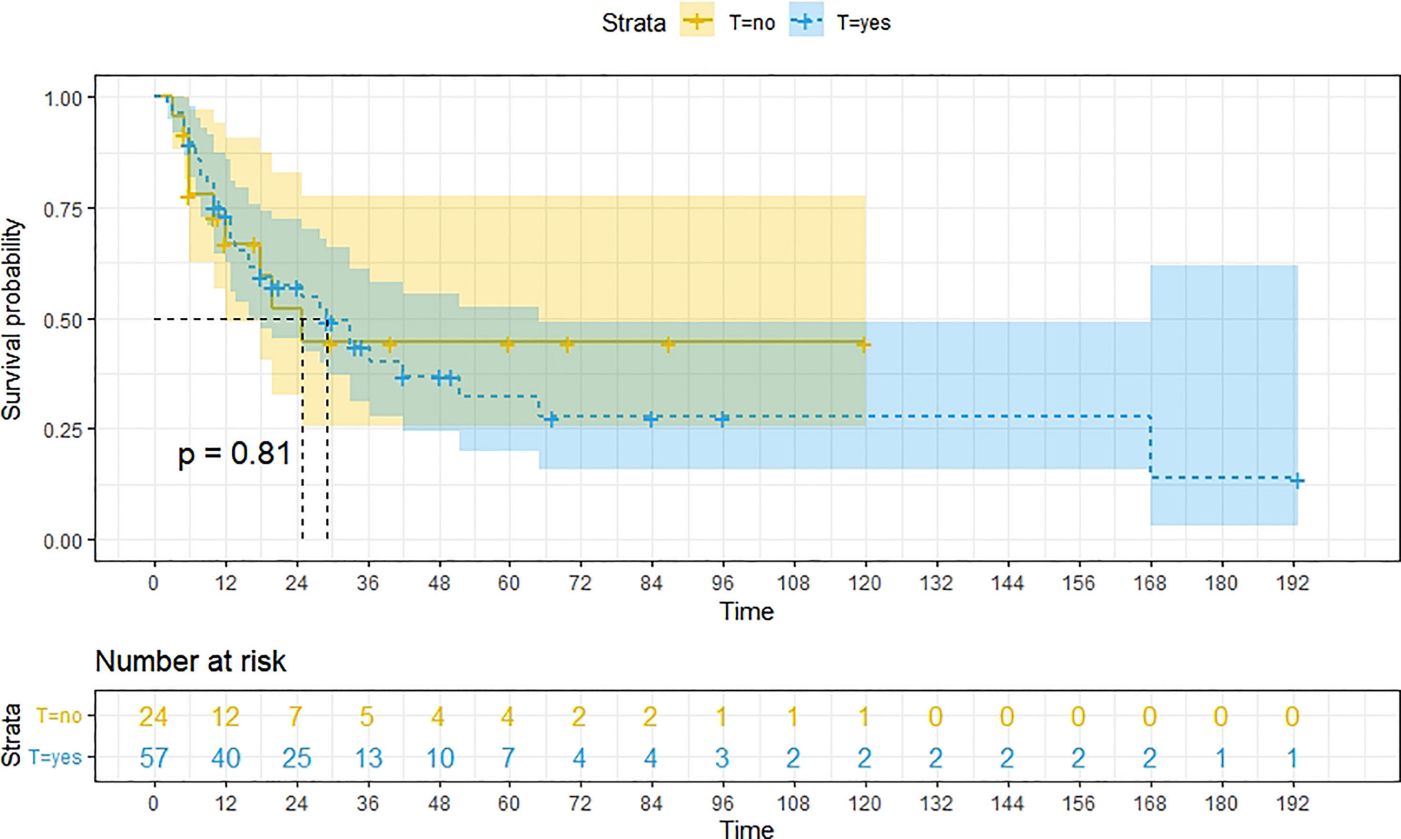
Kaplan-Meier survival curves for OS in patients of surgery (RH+TAH) with and without other treatments. There was little difference in median OS between these two groups.

**Figure 8:**
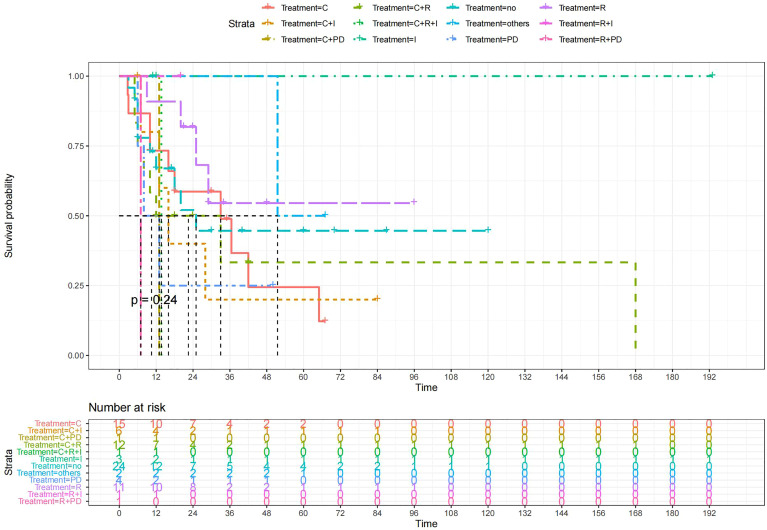
Kaplan-Meier survival curves for OS in patients of surgery (RH+TAH) plus various treatments vs. no other treatments. The median OS was significantly longer for patients who added chemotherapy and other treatments(oncolytic virus Rigvir^®^, argon laser, Argon heliumknife) than for those who added the remaining treatment modalities (Except for radiotherapy, Survival rates of this group at the 54m time point were higher for patients than other groups.) C: chemotherapy R: radiotherapy I: immunotherapy (interferon-γ or interleukin-2) PD : PD-1/PD-L1 Others: oncolytic virus Rigvir^®^, argon laser, Argon heliumknife+Sunitinib no:no other treatment.

**Figure 9:**
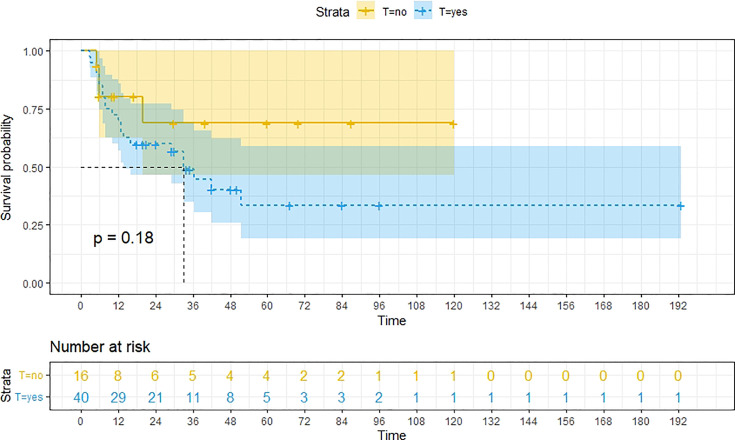
Kaplan-Meier survival curves for OS in patients of RH-based surgery with and without other treatments. Survival rates at the 24m, 36m, 48m and 60m time points were significantly higher for patients who did not add other treatment modalities than for those who did.

**Figure 10:**
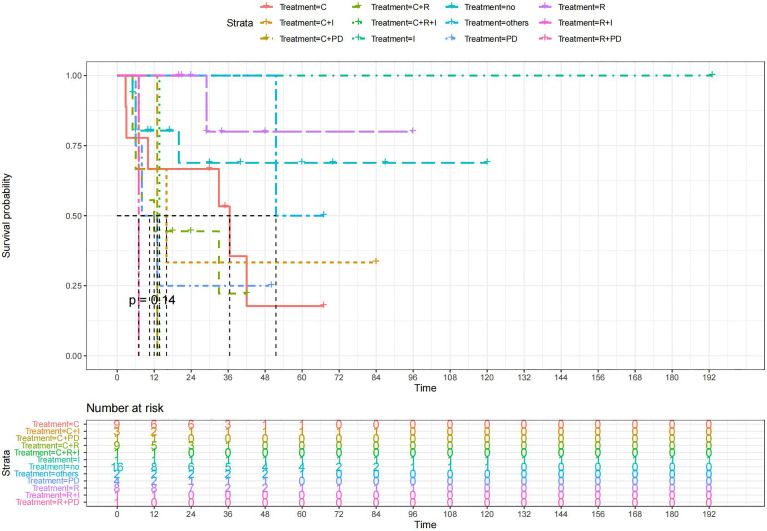
Kaplan-Meier survival curves for OS in patients of RH-based surgery plus various treatments vs. no other treatments. The median OS was significantly longer for patients who added chemotherapy and other treatments(oncolytic virus Rigvir^®^, argon laser, Argon heliumknife) than for those who added the remaining treatment modalities (Except for radiotherapy and no other treatment added, Survival rates of these two group at the 48m time point were significantly higher for patients than others.) C:chemotherapy R:radiotherapy I:immunotherapy(interferon-γ or interleukin-2) PD : PD-1/PD-L1 Others:oncolytic virus Rigvir^®^, argon laser, Argon heliumknife+Sunitinib no:no other treatment.

**Figure 11:**
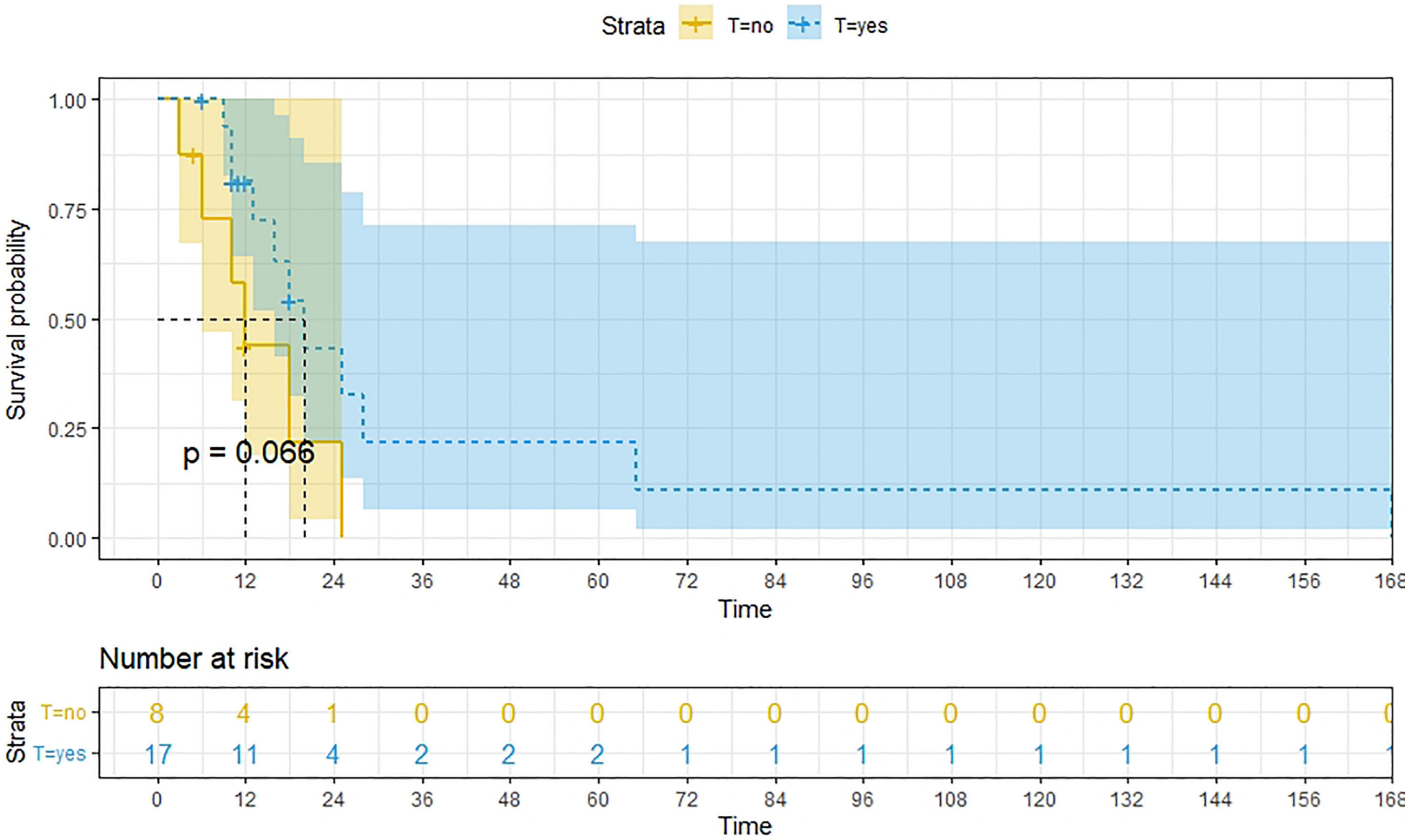
Kaplan-Meier survival curves for OS in patients of TAH-based surgery with and without other treatments. The median OS for them are 20m,12m respectively.

**Figure 12:**
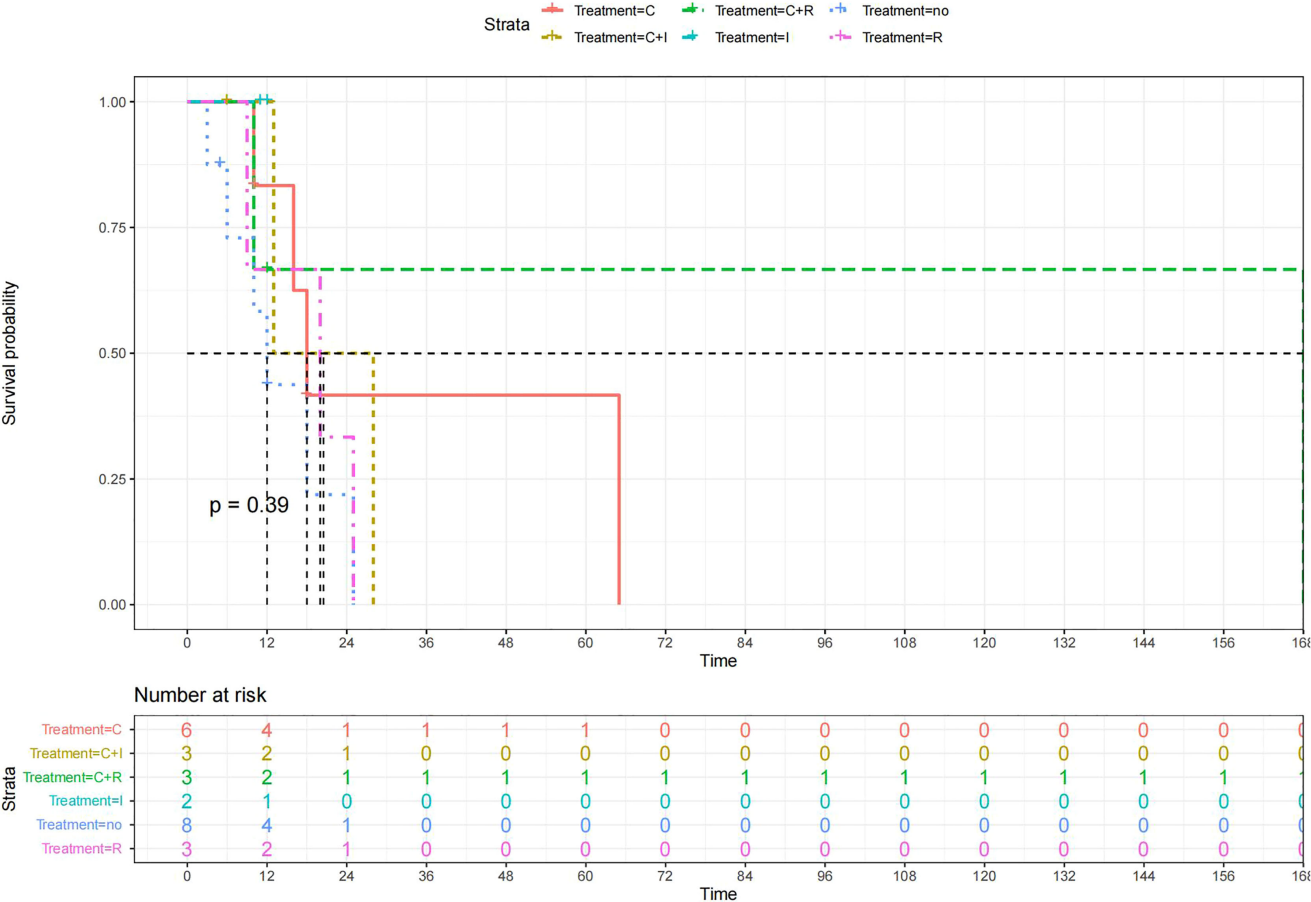
Kaplan-Meier survival curves for OS in patients of TAH-based surgery plus various treatments vs. no other treatments. The median OS was significantly longer for patients who added chemotherapy+radiotherapy than for those who added the remaining treatment modalities C:chemotherapy R:radiotherapy I:immunotherapy(interferon-γ or interleukin-2) PD : PD-1/PD-L1 Others:oncolytic virus Rigvir^®^,argon laser,Argon heliumknife+Sunitinib no:no other treatment.

A total of 99 patients had information on survival time and status, of whom 39 survived (5-193 months) while 60 died. Corresponding information was not available for 32 patients, whereas the survival status of 18 patients could not be determined owing to a lack of full text. We generated Kaplan–Meier survival curves to evaluate OS of the patients, and obtained a median OS of 18 months in the cohort (**Figure 2**). Next, we employed log-rank tests under stratified covariates (FIGO stage, Surgery or not, the extent of surgery, Lymphatic metastases or not, Lymphadenectomy or not, Supplementary treatment or not), to explore underlying factors that may affect patient prognosis. Results indicated that prognosis of patients significantly decreased with stage progression (P=0.00069; **Figure 3**). Notably, patients who did not have surgery had significantly worse prognosis compared to those who had RH-based and TAH-based surgery (P<0.0001), while those who had RH-based surgery had better prognosis than those who had TAH-based surgery (**Figure 4**). Moreover, patients with non-lymphatic metastases had higher median survival times than those with lymphatic metastases, albeit with no statistical significance (P=0.056; **Figure 5**). Patients who underwent lymphadenectomy exhibited better prognosis than those who did not undergo the procedure (P<0.0001; **Figure 6**). Next, we compared surgical results (RH+TAH, RH, TAH) with and without other treatments, and found that in patients who underwent surgery(RH+TAH), there was no statistically significant differences in patient prognosis between groups with or without the addition of other treatment modalities (P=0.81; **Figure 7**). However, patients who added chemotherapy and others treatments(oncolytic virus Rigvir^®^, argon laser, Argon heliumknife) had significantly longer median OS than those who added the remaining treatment modalities. The exception to this was in patients who added radiotherapy or immunotherapy, where survival rates at the 60m time point were significantly higher than others (**Figure 8**). Moreover, in patients who had RH-based surgery, we found survival rates at the 24m time point of those who did not add other treatments was higher than those who did (**Figure 9**; P=0.18). In order to exclude possible confounding effects due to inhomogeneous distribution of characteristics between patients with RH and patients with RH+T, we tested if the stage, LS, LM are the same (**Supplementary Table**). The results found no significant difference between the two groups for Stage, LS, but a significant difference for LM. Similarly, patients who had chemotherapy or other treatments (oncolytic virus Rigvir^®^, argon laser, Argon heliumknife) exhibited significantly better median OS than those who had the remaining treatments. The exception was observed in patients who underwent radiotherapy, immunotherapy or with no other treatment added, whose survival rates at the 54m were significantly higher than others (**Figure 10**; P=0.14). In patients who had TAH-based surgery, we found no statistically significant differences in prognosis between the group with or without addition of other treatment modalities. However, the former group had higher median survival times than the latter (**Figure 11**; P=0.066). Furthermore, patients who added chemotherapy+radiotherapy exhibited significantly longer OS than those who added the remaining treatment modalities. The median survival time for TAH-based patients with the addition of other treatment modalities was higher than for those who had only TAH surgery (**Figure 12**; P=0.39).

**Figure 13:**
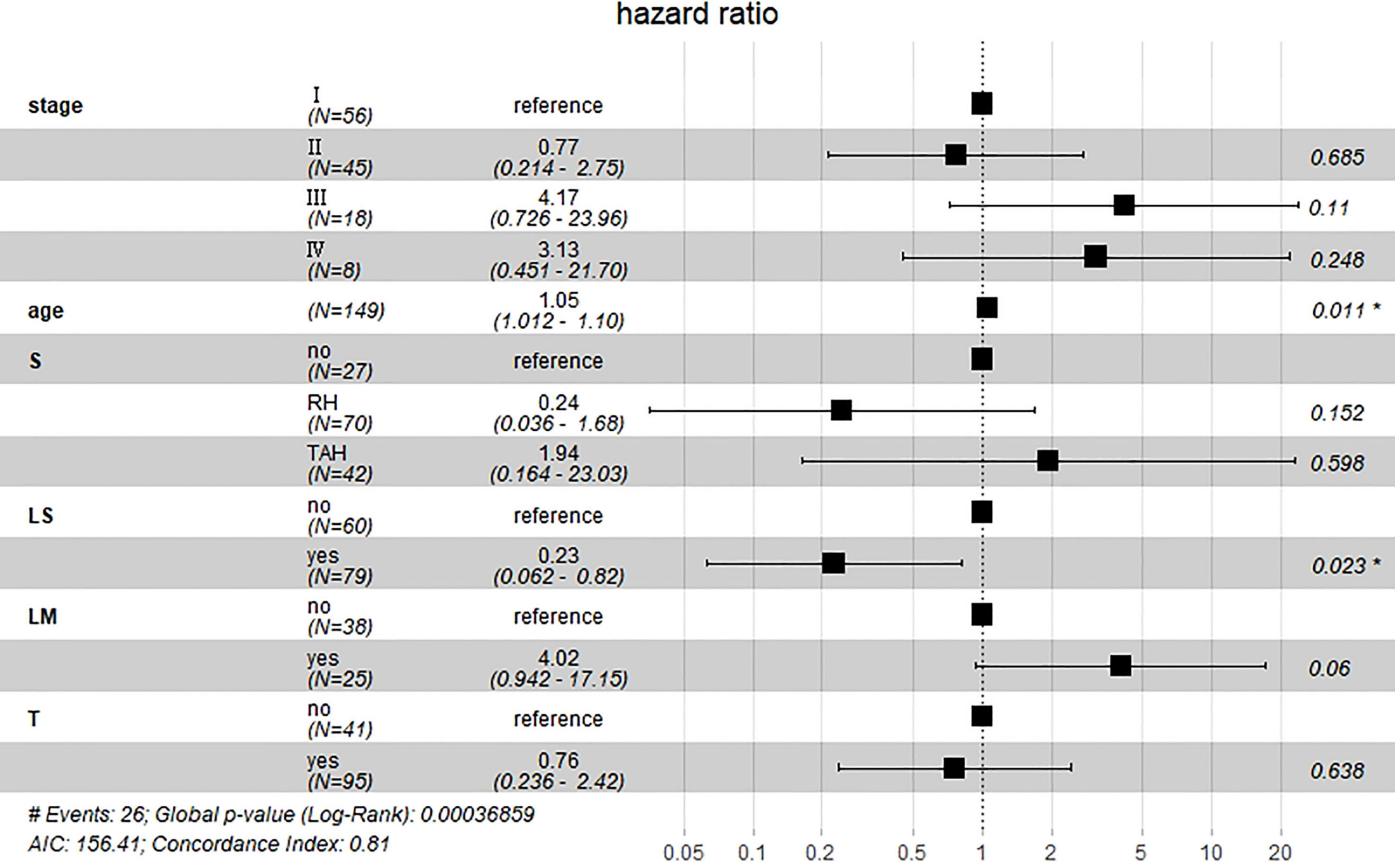
Multivariate Cox hazard model analysis for all patients. Age, stage III&IV, TAH, and lymph metastasis increased the risk of death, whereas RH and lymphadenectomy was associated with reduced risk of death. *means P<0.05.

**Figure 14:**
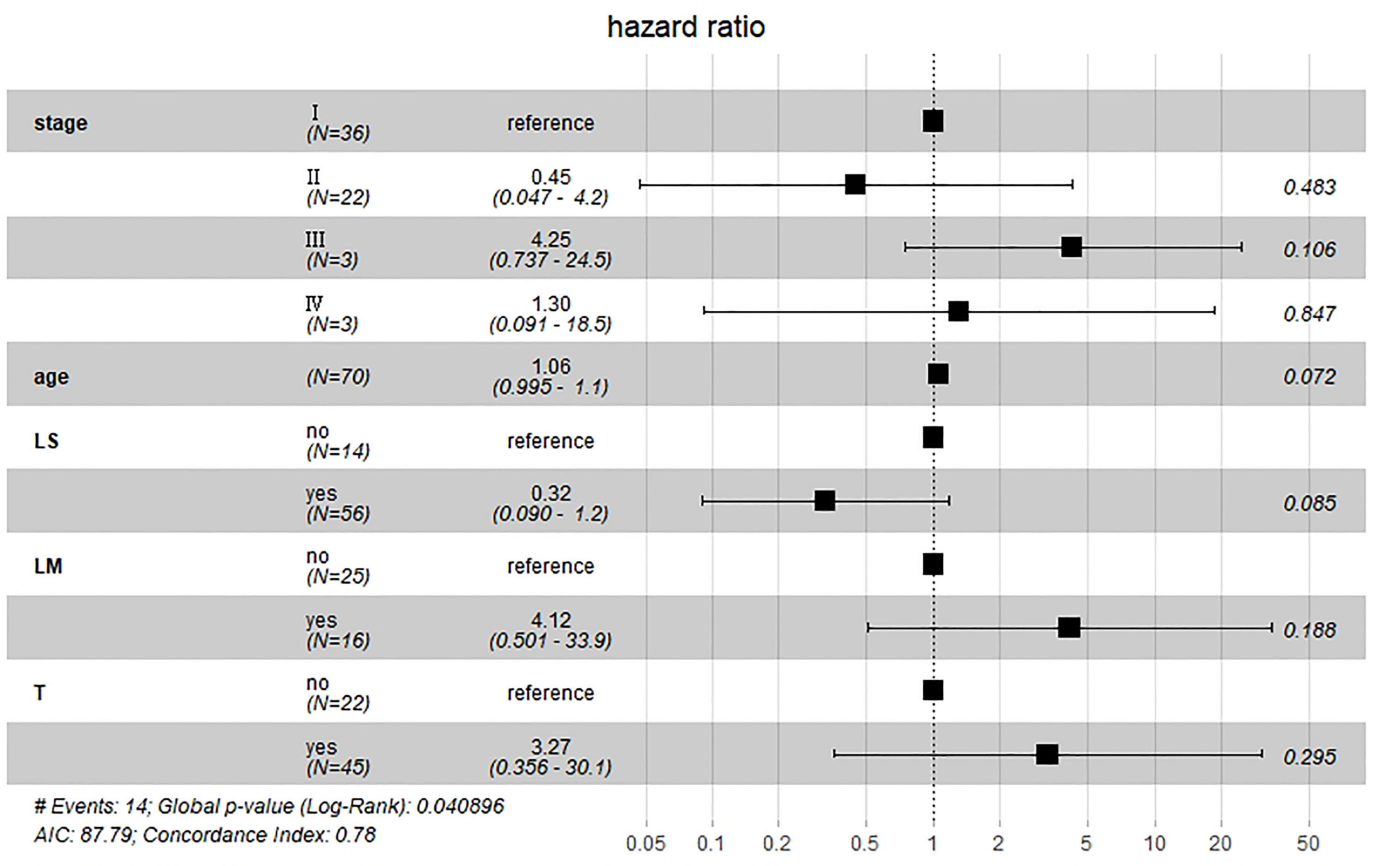
Multivariate Cox hazard model analysis for RH-based patients. Lymphadenectomy reduces the risk of death, while lymphatic metastases and complementary other treatments increase the risk of death.

**Figure 15:**
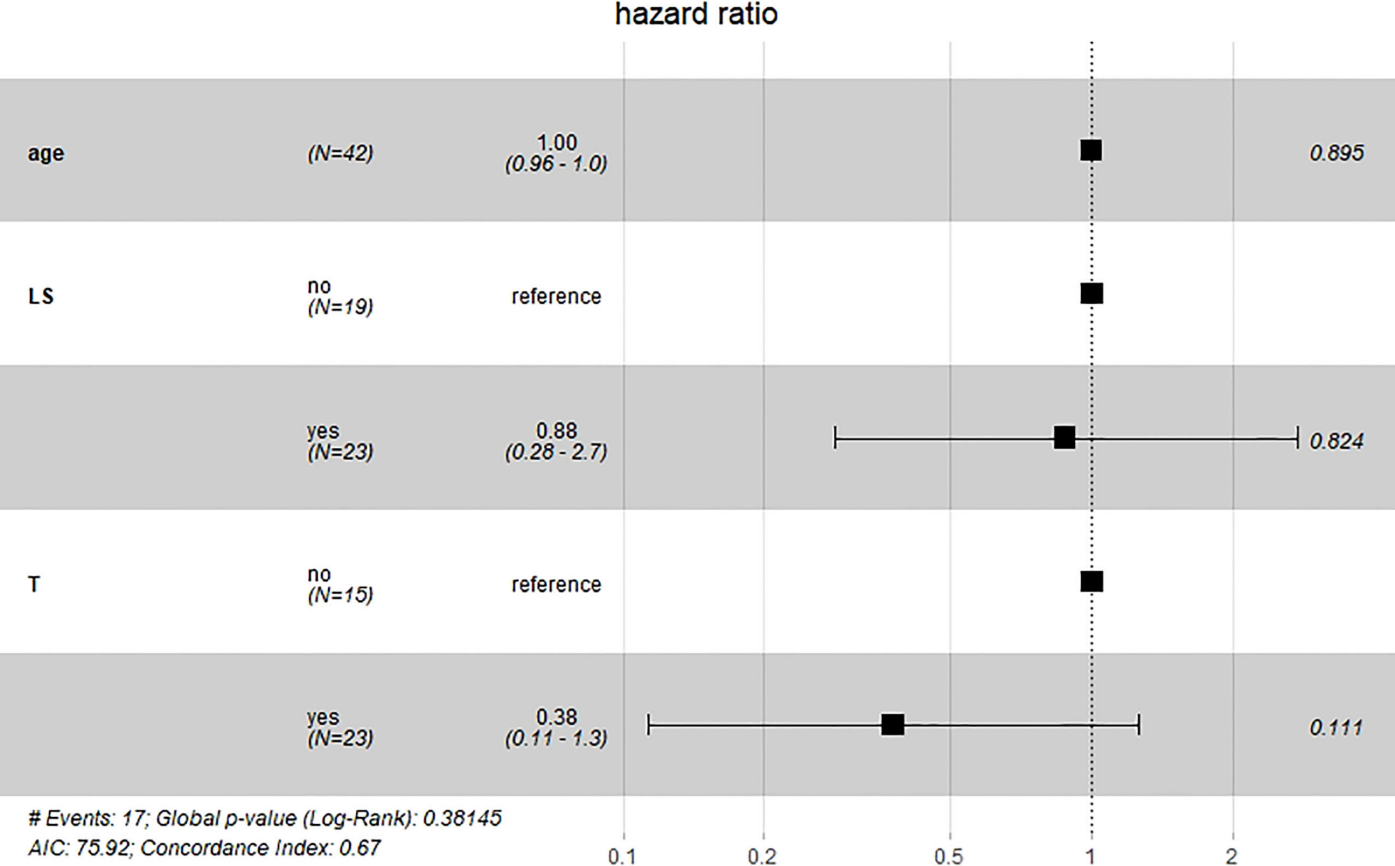
Multivariate Cox hazard model analysis for TAH-based patients. Lymphadenectomy seems to have little effect, while complementary treatment reduces the risk of death.

Results of multivariate Cox hazard model analysis revealed that age, stage III&IV, TAH and lymph metastasis increased the risk of death, whereas RH and lymphadenectomy was associated with reduced risk of death (**Figure 13**). In both the univariate and multifactorial cox regression risk models, only age and lymphatic resection showed consistency and could therefore be used as independent prognostic factors (**Table 2**). For patients who have undergone RH-based surgery, lymphadenectomy reduces the risk of death, while lymphatic metastases and complementary other treatments increase the risk of death (**Figure 14**). For patients who have undergone TAH-based surgery, lymphadenectomy seems to have little effect, while complementary treatment reduces the risk of death (**Figure 15**).

**Table 2:** Clinical factors effect on overall survival by univariate and multivariate cox proportional hazard regression analysis.

	Univariate analyzes		Multivariate analysis	
Characteristics	HR(95% CI)	P value	HR(95% CI)	P value
age	1.022(1.002-1.042)	0.0306	1.0546(1.01249-1.0984)	0.0105
Stage I	1		1	
II	1.826(0.9725-3.427)	0.061046	0.7676(0.21393-2.7545)	0.6850
III	4.765(2.1692-10.469)	0.000101	4.1698(0.72557-23.9643)	0.1095
IV	1.389(0.4026-4.792)	0.602981	3.1280(0.45096-21.6976)	0.2485
No Surgery	1		1	
RH	0.1523(0.07749-0.2993)	4.79e-08	0.2443(0.03559-1.6767)	0.1516
TAH	0.2966(0.14636-0.6011)	0.000745	1.9431(0.16396-23.0273)	0.5985
No lymphadenectomy	1		1	
lymphadenectomy	0.3143(0.1851-0.5336)	1.82e-05	0.2260(0.06244-0.8182)	0.0235
No lymph metastasis	1		1	
lymph metastasis	2.11(0.9717-4.583)	0.0591	4.0186(0.94152-17.1519)	0.0603
No treatments or only surgery	1		1	
Treatments other than surgery	1.429(0.741-2.757)	0.287	0.7562(0.23645-2.4186)	0.6376

## Discussion

Primary malignant melanoma of the cervix is an extremely rare disease. According to Norris and Taylor (129), cervical melanoma can be diagnosed based on four criteria, namely: presence of melanin in the normal cervical epithelium; absence of melanoma elsewhere in the body; junction changes in the cervix; and metastasis following a pattern of cervical cancer. The disease is characterized by poor patient prognosis, especially if it is not detected in time or treated correctly (95). Previous studies have shown that the 5-year OS for patients with this cancer is approximately 10% and many patients die within three years of diagnosis (87.5%) (13). However, the present study revealed contrasting results. as evidenced by a 5-year OS of 27% and death rate of 65% within 3 years. We attribute this to the continuous improvement in surgery and other treatment modalities. The main prognostic factor for cervical melanoma is the FIGO stage at the time of diagnosis (13). This is consistent with the results the present study, which revealed that median survival time gradually decreased with increasing FIGO stage.

Although no standard treatment modality has been developed for this condition, RH with pelvic lymph node dissection, partial vaginectomy remain the first-choice therapy for patients suitable for the procedure (130, 131). Results of the present study indicated that RH-based surgery did improve patient survival times, which were significantly better than those of patients who had TAH-based surgery and those who did not have surgery. However, a total hysterectomy was performed in some cases. Adjuvant pelvic radiotherapy may be considered for patients with positive surgical margins, parametrial involvement or histologically positive nodes. On the other hand, patients who are not suitable for radical surgery may be subjected to definitive external pelvic radiotherapy with/without brachytherapy, primarily for palliative purposes (131). Although our results were consistent with this conclusion, we believe that adjuvant other treatments are counterproductive in patients who have undergone RH-based surgery. This conclusion is contrary to common sense. It may be related to the following reasons:1.The difference in LM numbers between the two groups was significant, while the median survival time was significantly lower for patients with lymph node metastases than for those without; 2.the sample size of patients supplemented with other treatments was not large enough; 3. each case came from a different study unit, so there was some variation in the quality of the procedure even for radical surgery. If the extent of surgery is inadequate, for example TAH, adjuvant other treatments may improve their median survival time.

Immunotherapy, particularly immune checkpoint inhibitors, has shown great promise in cancer treatment (132), whereas immunotherapy based on immune checkpoint blockade is efficacious in treating melanoma (133). Although some studies have suggested that anti-PD-1 is associated with better OS compared to anti-CTLA4 in advanced/recurrent female genital tract melanoma (134), our results demonstrated that PD agents were not superior to the other adjuvant treatment modalities in patients with PMMC. This may be due to the small sample size of patients enrolled in this study.

In summary, this is the first pooled analysis including 149 cases of primary cervical melanoma. We found that patients who underwent RH-based surgery, those with non-lymph nodes metastatic and those who underwent lymphadenectomy had significantly higher survival rates. Based on the results of the analysis, the addition of other treatment options for patients who undergoing RH-based surgery is subject to further study, but for those who had TAH-based surgery, the addition of other treatments to prolong median survival may be considered. Notably, age and lymphadenectomy were associated with increased and reduced risk of death in these patients, respectively. Although there was no statistically significant difference, stage III&IV, TAH and lymph metastasis increased the risk of death, whereas RH was associated with reduced risk of death. For patients who have undergone RH-based surgery, lymphadenectomy reduces the risk of death, while lymphatic metastases and complementary other treatments increase the risk of death. For patients who have undergone TAH-based surgery, lymphadenectomy seems to have little effect, while complementary treatment reduces the risk of death. Future collaborative epidemiological studies are needed to further validate these findings. Therefore, *via* summarizing previous reports, the recommended treatment procedure for PMMC are radical hysterectomy and lymphadenectomy. The addition of other treatment options for patients who undergoing RH-based surgery need further study.

## Data Availability Statement

The datasets generated during and/or analyzed during the current study are available from the corresponding author on reasonable request.

## Ethics Statement

Ethical review and approval was not required for the study on human participants in accordance with the local legislation and institutional requirements. Written informed consent for participation was not required for this study in accordance with the national legislation and the institutional requirements.

## Author Contribution

HC wrote the manuscript. AM and AF participated in the search strategy development. AM assisted in acquisition, analysis, or interpretation of data for the current work. HC prepared figures and tables. HW contributed to study concept and design. HC and MH double-checked the data and corrected the error in the tables. All authors contributed to critical manuscript revision.

## Funding

This study was financially supported by Annual Science and Technology Steering Plan Project of Zhuzhou and Sichuan Science and Technology 259 Program (2021YFS0126).

## Conflict of Interest

The authors declare that the research was conducted in the absence of any commercial or financial relationships that could be construed as a potential conflict of interest.

## Publisher’s Note

All claims expressed in this article are solely those of the authors and do not necessarily represent those of their affiliated organizations, or those of the publisher, the editors and the reviewers. Any product that may be evaluated in this article, or claim that may be made by its manufacturer, is not guaranteed or endorsed by the publisher.
