# Supplementary material for: Primary Malignant Melanoma of the Cervix: An Integrated Analysis of Case Reports and Series
Source: Front Oncol. 2022 Jun 22;12:913964. doi: 10.3389/fonc.2022.913964 (PMC9258497; doi:10.3389/fonc.2022.913964)
Supplement: Supplementary file 1 [file Table_1.docx]

|  | RH | RH+T | X^2^ | P |
| --- | --- | --- | --- | --- |
| StageⅠ | 13 | 21 |  |  |
| StageⅡ | 7 | 14 |  |  |
| StageⅢ | 0 | 3 |  |  |
| StageⅣ | 0 | 3 | 3.388 | 0.336 |
| LS(yes) | 19 | 37 |  |  |
| LS(no) | 6 | 8 | 0.389 | 0.533 |
| LM(yes) | 2 | 14 |  |  |
| LM(no) | 12 | 13 | 5.468 | 0.041 |

Supplementary table Comparison of clinical features between the Rh and RH+T groups

Used tests: Chi-square test
